# Cemented total hip arthroplasty reduces early complications: a Japanese nationwide propensity-matched study

**DOI:** 10.1007/s00402-026-06328-x

**Published:** 2026-05-02

**Authors:** Hidetatsu Tanaka, Kunio Tarasawa, Yu Mori, Kazuyoshi Baba, Hiroaki Kurishima, Kiyohide Fushimi, Toshimi Aizawa, Kenji Fujimori

**Affiliations:** 1 Department of Orthopaedic Surgery, Tohoku University Graduate School of Medicine, Sendai, Japan; 2 Department of Medical Information Technology Center, Tohoku University Hospital, Sendai, Japan; 3 Department of Orthopaedic Surgery, Japanese Red Cross Sendai Hospital, Sendai, Japan; 4 Department of Health Policy and Informatics, Institute of Science Tokyo, Tokyo, Japan; 5 Professor Emeritus, Tohoku University, Sendai, Japan

**Keywords:** Total hip arthroplasty, Cemented fixation, Cementless fixation, Complication, Diagnosis Procedure Combination, Propensity score analysis

## Abstract

**Introduction:**

The optimal fixation method in total hip arthroplasty (THA) remains under debate. While cemented fixation has been associated with a lower risk of periprosthetic fracture, uncemented fixation predominates in Japan. This study aimed to compare early postoperative complications between cemented and uncemented fixation in elective THA using a nationwide inpatient database.

**Materials and methods:**

We identified 198,102 patients aged ≥ 65 years who underwent primary THA for osteoarthritis, osteonecrosis, or rheumatoid arthritis between December 2011 and March 2023 from the Japanese Diagnosis Procedure Combination (DPC) database. After 1:1 propensity score matching for age, sex, body mass index (BMI), and Charlson Comorbidity Index, 36,859 patients were included in each fixation cohort. Surgical and medical complications, and in-hospital mortality were compared using multivariate logistic regression.

**Results:**

Cemented fixation was associated with a significantly lower risk of periprosthetic fracture (odds ratio [OR], 0.40; 95% confidence interval [CI], 0.30–0.53; *p* < 0.001), blood transfusion (OR, 0.76; 95% CI, 0.74–0.78; *p* < 0.001), and deep vein thrombosis (OR, 0.79; 95% CI, 0.74–0.84; *p* < 0.001). There were no statistically significant differences based on the predefined threshold (*p* < 0.001) in dislocation, infection, pulmonary embolism, cardiac or cerebrovascular events, or in-hospital mortality between fixation types, although a trend toward higher in-hospital mortality in the cemented group was observed.

**Conclusions:**

Cemented THA was associated with reduced rates of periprosthetic fracture, transfusion, and deep vein thrombosis without increasing other perioperative or medical complications. These findings suggest that cemented fixation may be associated with favorable short-term outcomes in selected patients.

**Supplementary Information:**

The online version contains supplementary material available at 10.1007/s00402-026-06328-x.

## Introduction

Total hip arthroplasty (THA) has been described as one of the most successful surgical procedures of the century, providing substantial pain relief and functional recovery for patients with degenerative hip disease [14]. Nevertheless, the optimal method of femoral fixation—cemented or uncemented—remains a subject of ongoing debate [2, 7, 11, 19, 23, 26]. Numerous registry analyses have demonstrated that cemented femoral fixation is associated with a lower incidence of periprosthetic femoral fracture compared with uncemented fixation [2, 26]. Despite this consistent evidence, uncemented fixation has become the predominant technique in many countries, including Japan, where approximately 85% to 90% of primary THAs are performed using uncemented stems [22]. This discrepancy—often referred to as the “Japanese paradox” in THA—underscores the need for evidence derived from Japan’s clinical environment [22]. The rationale for this preference remains unclear, but it may reflect surgeons’ perceptions that uncemented fixation provides superior long-term biological fixation, requires less surgical manipulation, and is better suited to the relatively younger Japanese THA population, rather than conclusive evidence of improved outcomes.

Beyond periprosthetic fractures, several other surgical and medical complications have been compared between cemented and uncemented fixation. Historical concerns regarding bone cement implantation syndrome (BCIS) in the 1970s led to a global shift toward uncemented fixation [1, 5, 9, 23]. However, large European registry studies have shown that perioperative and long-term mortality rates are similar between the two fixation types [2, 3, 5, 15, 23]. Infection has also been an area of controversy. Although antibiotic-loaded cement theoretically provides local antimicrobial protection, most registry studies and meta-analyses have not demonstrated a significant advantage of cemented fixation in preventing periprosthetic joint infection [15, 17]. Conversely, several North American studies have reported slightly higher short-term infection rates in cemented THA, possibly reflecting differences in patient selection and comorbidity burden rather than fixation itself [4, 19]. Dislocation remains another major complication following THA. While some studies have reported comparable dislocation rates between cemented and uncemented fixation [15], others have found a higher incidence associated with cemented stems [4]. These discrepancies likely arise from differences in patient demographics, implant design, and surgical approach among studies conducted in diverse healthcare systems.

To date, no nationwide study has comprehensively compared perioperative outcomes between cemented and uncemented THA in Japanese patients. The aim of this study was to determine whether cemented fixation is associated with early postoperative complications compared with uncemented fixation in elective THA for osteoarthritis, osteonecrosis of the femoral head, and rheumatoid arthritis, using the Japanese Diagnosis Procedure Combination (DPC) national inpatient database. Specifically, we examined (1) hip- and surgery-related complications, (2) medical complications, and (3) in-hospital mortality, adjusting for baseline differences between the two groups.

## Patients and methods

### Study design and setting

This retrospective cohort study used data from the Japanese Diagnosis Procedure Combination (DPC) database, in accordance with the principles of the Declaration of Helsinki. Ethical approval was obtained from the Institute of Science Tokyo (Approval No. M2000-788) and the Tohoku University Graduate School of Medicine (Approval Nos. 2021-1-1082 and 2024-1-1026). The dataset covered the period from December 2011 to March 2023 and included clinical information from approximately 1100 hospitals participating in the DPC system across Japan. The database provides anonymised, patient-level data including demographics, diagnoses (International Classification of Diseases, 10th Revision; ICD-10), admission and discharge dates, surgical and non-surgical procedures, comorbidities, and in-hospital complications. The DPC database covers approximately 70% of acute-care hospital beds in Japan and has been widely used and validated for clinical and epidemiological research, although some degree of coding inaccuracy is inherent to administrative data.

### Participants and data selection

Patients aged ≥ 65 years who underwent unilateral total hip arthroplasty (THA) for osteoarthritis of the hip (ICD-10 codes M160–M169), osteonecrosis of the femoral head (M8705, M8715, M8725, M8735, M8785, and M8795), or rheumatoid arthritis (M0695, M0690, M0685, M0605, M0595, M0585, M0586, M1315) were included. Patients who underwent THA for trauma-related conditions such as proximal femoral or acetabular fractures were excluded. Between December 2011 and March 2023, a total of 198,102 eligible patients were identified. The analysis focused on resource-intensive cases as defined by the DPC system, reflecting the real-world clinical practice of THA in Japan.

**Fig. 1:**
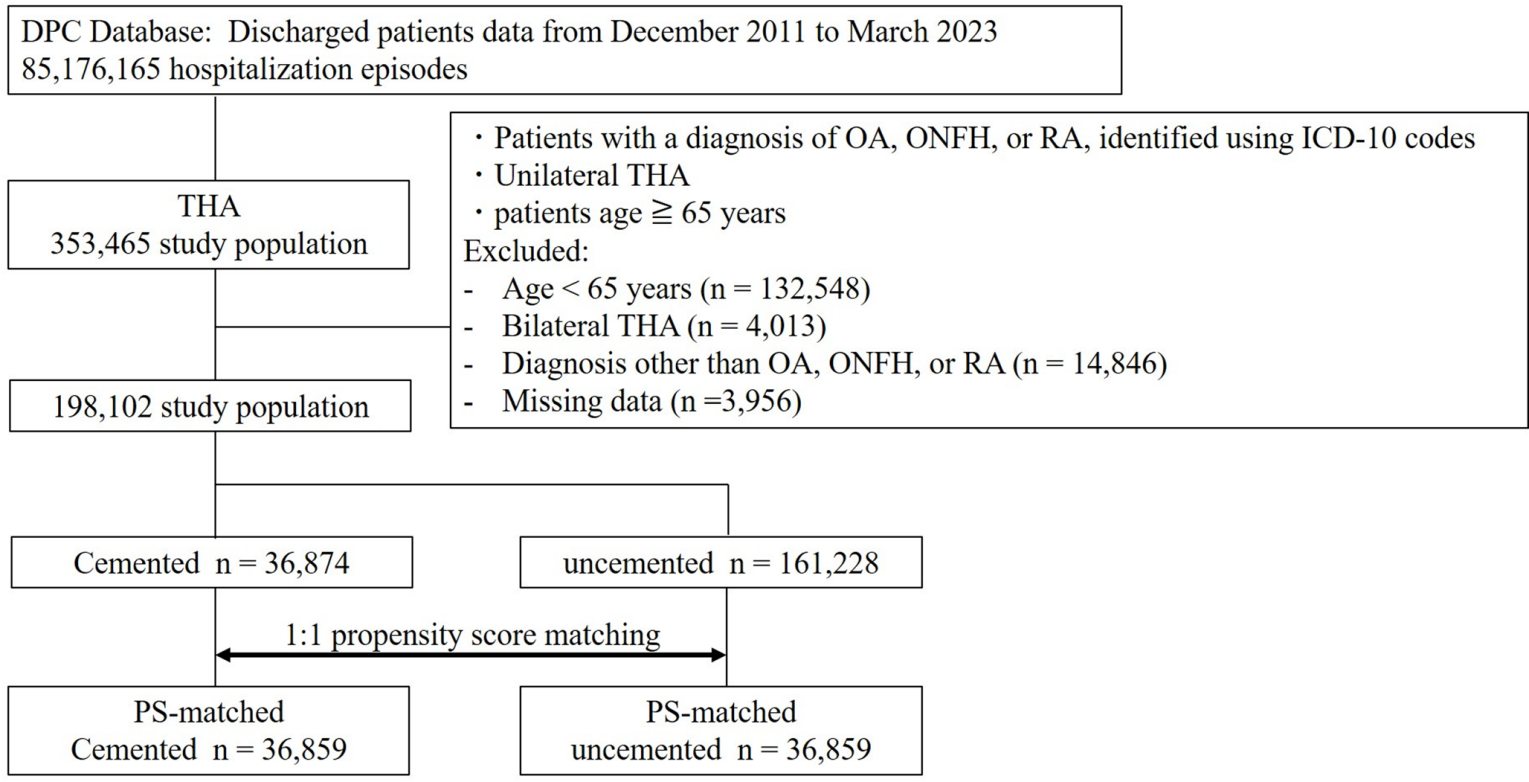
Study flow chart

Propensity score (PS) matching was performed in a 1:1 ratio using age, sex, body mass index (BMI), and Charlson Comorbidity Index (CCI) as covariates. The discriminative ability of the PS model was assessed using the C-statistic, and nearest-neighbor matching without replacement was conducted with a caliper width of 0.2 times the standard deviation of the PS. After matching, 36,859 patients were included in each of the cemented and uncemented THA cohorts. The C-statistic was 0.866, and all covariates achieved standardised mean differences (SMD) of < 0.1, indicating adequate balance between groups (Table 1, Fig. 1). We restricted the analysis to patients aged ≥ 65 years to improve comparability between fixation groups and to focus on a population in whom fixation choice is clinically most relevant.

**Table 1:** Baseline demographic data

	Before PS matching			After PS matching		
	Cemented	Uncemented	*P*-value	Cemented	Uncemented	SMD
n	36,874	161,228		36,859	36,859	
Age	74.7 ± 6.3	74.2 ± 6.1	< 0.001※	74.7 ± 6.3	74.7 ± 6.3	0.001
Gender (%)						
Men	5494 (14.9)	25,574 (15.9)	< 0.001	5491 (14.9)	5216 (14.3)	0.023
Women	31,380 (85.1)	135,654 (84.1)		31,368 (85.1)	31,643 (85.9)	
BMI	23.7 ± 3.8	23.9 ± 3.8	< 0.001※	23.7 ± 3.8	23.6 ± 3.7	0.007
CCI	0.57 ± 0.91	0.51 ± 0.85	< 0.001	0.57 ± 0.91	0.55 ± 0.90	0.015
Length of hospital stay	27.7 ± 16.8	27.9 ± 16.9	0.136	27.7 ± 16.8	28.3 ± 16.9	0.033
Use of TXA	20,234 (54.9)	93,217 (57.8)	< 0.001	20,226 (54.9)	21,128 (57.3)	0.049
Post-operative Anticoagulate agent (%)	28,532 (77.4)	127,261 (78.9)	< 0.001	28,519 (77.4)	29,115 (79.0)	0.039

Age, BMI, CCI, waiting days for surgery, and length of hospital stay are shown as mean ± standard deviation

P-values of < 0.001 are considered significant by the Student-t test and χ^2^ test

BMI: Body Mass Index, CCI; Charlson Comorbidity Index, SMD; Standardized Mean Difference, TXA; Tranexamic acid

※ Student-t test

### Exposure and outcomes

Patients were categorised into cemented or uncemented groups according to perioperative medication codes recorded on the day of surgery. Primary surgical outcomes included postoperative dislocation, surgical site infection, periprosthetic fracture, wound dehiscence, mechanical loosening, blood transfusion, and reoperation. Medical complications included hospital-acquired pneumonia, deep vein thrombosis (DVT), pulmonary embolism (PE), cardiac events, cerebrovascular events, acute renal failure, sepsis, and in-hospital mortality.

### Statistical analysis

Categorical variables were compared using the chi-squared test and continuous variables using Student’s *t*-test. Univariate and multivariate logistic regression analyses were performed to assess the association between cement use and each complication, adjusting for covariates. Data are presented as mean ± standard deviation (SD) or odds ratio (OR) with 95% confidence interval (CI). Statistical significance was defined as *p* < 0.001 to account for the large sample size and multiple comparisons, thereby reducing the risk of type I error. This threshold was not based on a formal multiplicity correction method but was selected as a conservative criterion commonly used in large administrative database studies. Results with p-values between 0.001 and 0.05 were considered exploratory and interpreted as trends rather than statistically significant findings. SMD < 0.1 indicated adequate balance. All regression analyses were conducted within the propensity score–matched cohort. Standard logistic regression models were used without accounting for matched pairs, as covariate balance was achieved after matching. The regression analyses were performed to further adjust for residual confounding. This approach was not intended as a doubly robust estimation but rather as an additional adjustment within the matched cohort. Although conditional logistic regression could account for matched pairs, standard logistic regression was used after confirming adequate covariate balance (SMD < 0.1), which is a commonly adopted approach in large-scale database studies [21, 31]. All analyses were performed using JMP version 18.0 (SAS Institute, Cary, NC, USA).

## Results

### Surgical complications

Cemented fixation was associated with a significantly lower risk of periprosthetic fracture (OR, 0.40; 95% CI 0.30–0.53; *p* < 0.001) and blood transfusion (OR, 0.76; 95% CI, 0.74–0.78; *p* < 0.001). No statistically significant association was found based on the predefined threshold (*p* < 0.001) between cemented fixation and dislocation (OR, 1.29; 95% CI, 1.01–1.65; *p* = 0.040), infection (OR, 0.81; 95% CI, 0.68–0.96; *p* = 0.014), wound dehiscence (OR, 1.01; 95% CI, 0.65–1.58; *p* = 0.954), mechanical loosening (OR, 0.81; 95% CI, 0.43–1.53; *p* = 0.504), or reoperation (OR, 0.88; 95% CI, 0.73–1.06; *p* = 0.167) (Table 2); however, dislocation and infection showed p-values between 0.001 and 0.05 and were interpreted as exploratory trends rather than statistically significant findings.

**Table 2:** Association between use of cement and surgical complications and reoperation

Complications	*n*	Univariate analysis			Multivariable analysis			
		OR	95% CI	*P*-value	OR	95% CI	χ^2^ statics	*P*-value
Dislocation	561	1.086	0.920 to 1.283	0.351	1.290	1.012 to 1.646	4.225	0.040
Infection	559	0.808	0.683 to 0.955	0.014	0.809	0.683 to 0.959	5.986	0.014
Periprosthetic fracture	251	0.370	0.280 to 0.489	< 0.001	0.395	0.295 to 0.528	43.32	< 0.001
Wound dehiscence	79	0.975	0.627 to 1.516	1.000	1.013	0.650 to 1.579	0.003	0.954
Mechanical loosening	40	0.739	0.395 to 1.383	0.430	0.805	0.425 to 1.525	0.446	0.504
Transfusion	41,921	0.761	0.734 to 0.783	< 0.001	0.760	0.738 to 0.783	338.0	< 0.001
Reoperation	1030	0.868	0.767 to 0.981	0.026	0.875	0.725 to 1.057	1.913	0.167

P-values of < 0.001 are considered significant by the χ^2^ test. P-values between 0.001 and 0.05 were considered exploratory

OR; Odds Ratio, CI; Confidence Interval

### Medical complications

Cemented fixation was associated with a significantly lower risk of DVT (OR, 0.79; 95% CI, 0.74–0.84; *p* < 0.001). No statistically significant differences were found based on the predefined threshold (*p* < 0.001) in the incidence of hospital-acquired pneumonia, PE, cardiac events, cerebrovascular events, acute renal failure, sepsis, or in-hospital mortality. However, in-hospital mortality showed a p-value between 0.001 and 0.05 (OR, 1.96; 95% CI, 1.07–3.60; *p* = 0.026) and was interpreted as an exploratory trend rather than a statistically significant finding (Table 3).

**Table 3:** Association between use of cement and medical complications

Complications	*n*	Univariate analysis			Multivariable analysis			
		OR	95% CI	*P*-value	OR	95% CI	χ^2^ statics	*P*-value
Hospital-acquired pneumonia	142	1.058	0.761 to 1.471	0.801	1.042	0.748 to 1.452	0.058	0.809
DVT	3882	0.789	0.739 to 0.842	< 0.001	0.790	0.741 to 0.844	50.53	< 0.001
PE	160	0.839	0.614 to 1.145	0.304	0.841	0.615 to 1.150	1.184	0.277
Cardiac event	14	2.500	0.784 to 7.973	0.180	2.252	0.697 to 7.278	2.008	0.157
Cerebrovascular event	188	1.022	0.767 to 1.360	0.942	1.015	0.762 to 1.353	0.011	0.917
Acute renal failure	20	1.000	0.416 to 2.403	1.000	0.991	0.411 to 2.390	0.000	0.984
Sepsis	435	0.760	0.628 to 0.919	0.005	0.765	0.632 to 0.925	7.692	0.006
Mortality during hospitalization	48	2.001	1.098 to 3.647	0.029	1.957	1.065 to 3.596	4.937	0.026

OR; Odds Ratio, CI; Confidence Interval, DVT; Deep Vein Thrombosis, PE; Pulmonary Embolism

P-values of < 0.001 are considered significant by the χ^2^ test. P-values between 0.001 and 0.05 were considered exploratory

### Predictors of complications

Multivariate logistic regression identified several independent predictors of surgical and medical complications (Table 4). For periprosthetic fracture, older age (OR, 1.03; 95% CI, 1.01–1.05; *p* < 0.001) and female sex (OR, 2.66; 95% CI, 1.57–4.50; *p* < 0.001) were significant risk factors, while the use of bone cement was strongly protective (OR, 0.37; 95% CI, 0.28–0.49; *p* < 0.001). BMI (*p* = 0.668) and CCI (*p* = 0.034) were not significant at the predetermined threshold. For blood transfusion, older age (OR, 0.98; 95% CI, 0.98–0.99; *p* < 0.001), female sex (OR, 1.16; 95% CI, 1.11–1.21; *p* < 0.001), lower BMI (OR, 0.99; 95% CI, 0.98–1.02; *p* < 0.001), and lower CCI (OR, 0.94; 95% CI, 0.93–0.96; *p* < 0.001) were associated with an increased likelihood of transfusion. The use of bone cement was also associated with a reduced risk (OR, 0.76; 95% CI, 0.74–0.78; *p* < 0.001). For DVT, female sex (OR, 0.77; 95% CI, 0.69–0.85; *p* < 0.001), higher BMI (OR, 1.01; 95% CI, 1.01–1.02; *p* = 0.002), and the use of bone cement (OR, 0.79; 95% CI, 0.74–0.84; *p* < 0.001) were significant predictors. Age (*p* = 0.253) and CCI (*p* = 0.139) were not significantly associated with DVT. To further assess the robustness of our findings, we performed age-stratified multivariable analyses within the propensity score–matched cohort (65–74, 75–84, and ≥ 85 years). The association between cemented fixation and reduced risk of periprosthetic fracture remained consistent in the 65–74 and 75–84 age groups (Supplementary Tables S1 and S3). Similar patterns were observed for transfusion and DVT across age strata (Supplementary Tables S1–S6). Although estimates in the ≥ 85 years group were less precise due to smaller event numbers, no substantial heterogeneity in the direction of effect was observed. Sensitivity analyses using a narrower caliper width (0.05) yielded nearly identical results (Table 5), supporting the robustness of the findings.

**Table 4:** Multivariate logistic analysis of risk factors for surgical and medical complications

	Variable	OR	95% CI	χ^2^ statics	*P*-value
Periprosthetic fracture	Age	1.034	1.014 to 1.054	11.26	< 0.001
	Gender (women)	2.662	1.574 to 4.501	17.77	< 0.001
	BMI	0.993	0.959 to 1.026	0.184	0.668
	CCI	1.150	1.011 to 1.295	4.510	0.034
	Use of bone cement	0.371	0.281 to 0.491	54.60	< 0.001
Transfusion	Age	0.984	0.982 to 0.987	174.9	< 0.001
	Gender (women)	1.158	1.111 to 1.207	47.35	< 0.001
	BMI	0.985	0.981 to 1.015	56.26	< 0.001
	CCI	0.944	0.929 to 0.960	48.06	< 0.001
	Use of bone cement	0.761	0.739 to 0.784	333.6	< 0.001
DVT	Age	1.003	0.998 to 1.008	1.305	0.253
	Gender (women)	0.766	0.693 to 0.848	28.31	< 0.001
	BMI	1.014	1.005 to 1.023	9.870	0.002
	CCI	0.972	0.937 to 1.009	2.185	0.139
	Use of bone cement	0.790	0.740 to 0.843	50.64	< 0.001

P-values of < 0.001 are considered significant by the χ^2^ test

OR; Odds Ratio, CI; Confidence Interval, BMI: Boby Mass Index, CCI; Charlson Comorbidity Index, DVT; Deep Vein Thrombosis

**Table 5:** Multiivariate logistic regression analysis of postoperative surgical and medical complications associated with use of cement after propensity score matching using two different caliper widths for sensitivity analysis

Complications	Caliper Widths of 0.2			Caliper Widths of 0.05		
	OR	95% CI	*P*-value	OR	95% CI	*P*-value
Dislocation	1.290	1.012 to 1.646	0.040	1.277	1.001 to 1.630	0.049
Infection	0.809	0.683 to 0.959	0.014	0.812	0.685 to 0.962	0.016
Periprosthetic fracture	0.395	0.295 to 0.528	< 0.001	0.395	0.295 to 0.527	< 0.001
Wound dehiscence	1.013	0.650 to 1.579	0.954	0.986	0.631 to 1.541	0.950
Mechanical loosening	0.805	0.425 to 1.525	0.504	0.806	0.425 to 1.527	0.506
Transfusion	0.760	0.738 to 0.783	< 0.001	0.761	0.738 to 0.783	< 0.001
Reoperation	0.875	0.725 to 1.057	0.167	0.878	0.727 to 1.060	0.177
Hospital-acquired pneumonia	1.042	0.748 to 1.452	0.809	1.042	0.748 to 1.452	0.806
DVT	0.790	0.741 to 0.844	< 0.001	0.789	0.739 to 0.842	< 0.001
PE	0.841	0.615 to 1.150	0.277	0.841	0.615 to 1.150	0.278
Cardiac event	2.252	0.697 to 7.278	0.157	2.256	0.698 to 7.292	0.156
Cerebrovascular event	1.015	0.762 to 1.353	0.917	0.993	0.744 to 1.325	0.960
Acute renal failure	0991	0.411 to 2.390	0.984	0.991	0.411 to 2.389	0.983
Sepsis	0.765	0.632 to 0.925	0.006	0.768	0.635 to 0.929	0.006
Mortality during hospitalization	1.957	1.065 to 3.596	0.026	1.957	1.065 to 3.596	0.026

P-values of < 0.001 are considered significant by the χ2 test

OR; Odds Ratio, CI; Confidence Interval, DVT; Deep Vein Thrombosis, PE; Pulmonary Embolism

## Discussion

In this nationwide study using the Japanese DPC database, we compared early postoperative outcomes between cemented and uncemented THA. After rigorous propensity score matching, cemented fixation was not associated with increased overall complication risk but showed distinct outcome patterns across specific complications. This large-scale analysis based on more than 90,000 matched patients represents one of the largest nationwide evaluations of THA fixation methods in Japan, where uncemented fixation accounts for approximately 80% of all procedures. The Japanese DPC database serves as a valuable resource for large-scale cohort studies in orthopaedics, including research on hip fractures [21, 30, 31]. The major strength of this study lies in the inclusion of contemporary nationwide data, adjustment for major confounders, and focus on short-term in-hospital outcomes, which complement existing registry-based evidence from Europe and North America [2, 7, 17, 26, 29, 32].

Consistent with previous registry and database studies, cemented fixation markedly reduced the risk of periprosthetic fracture compared with uncemented fixation. The Swedish Arthroplasty Register reported an OR of 2.40 for fracture with uncemented fixation, highlighting the protective effect of cement use [2]. Similarly, analysis of the American Joint Replacement Registry demonstrated that cemented stems were associated with a 54–66% lower risk of periprosthetic fracture–related revision [26]. Notably, elderly Japanese patients often have lower bone mineral density compared to their Western counterparts [28], increasing their susceptibility to press-fit–related fractures. Bone integration at the implant interface requires both mechanical stability and an optimal biological environment. Key factors include mesenchymal stem cell differentiation into osteoblasts and an appropriately regulated inflammatory response, both essential for effective bone healing [10, 20]. Biomechanically, the cement mantle provides uniform load transfer and minimizes stress risers, which are more pronounced in uncemented stems relying on press-fit fixation, especially in elderly patients with osteoporotic bone. Given the global trend of increasing periprosthetic fracture incidence and the aging population, our findings support reconsideration of cemented fixation in selected older patients with compromised bone quality. On the other hand, recent reports have suggested that the use of collared uncemented stems may mitigate the risk of periprosthetic fracture to a level comparable with that of cemented stems [16, 24], several recent clinical studies have further demonstrated that collared uncemented stems reduce the risk of early periprosthetic femoral fracture by approximately three- to fourfold compared with collarless designs [8, 25], further research is desirable.

Periprosthetic joint infection remains a devastating complication after THA, and the influence of fixation method has been debated. Theoretically, antibiotic-loaded bone cement provides local antimicrobial prophylaxis. However, recent large-scale analyses have yielded conflicting results. The current study observed no significant difference in infection rates between fixation types, consistent with European registry data showing comparable infection risks regardless of cement use [5, 15, 33]. Although not reaching our predefined significance threshold (*p* < 0.001), a trend toward a lower risk of infection in the cemented group (OR 0.81; 95% CI, 0.68–0.96; *p* = 0.014) was observed, which may be clinically relevant and warrants further investigation. Conversely, a U.S. national database study reported higher early infection rates in cemented THA (4.5% vs. 0.8%) [19]. This discrepancy may reflect differences in patient characteristics: cemented fixation is typically chosen for older or more comorbid patients, who inherently have higher infection susceptibility [11]. Collectively, these findings suggest that host and perioperative factors more strongly drive infection risk than by fixation method itself.

In terms of dislocation, our analysis found no significant difference between cemented and uncemented fixation. However, although not meeting our predefined significance threshold, a trend toward an increased risk of dislocation in the cemented group was observed (OR 1.29; 95% CI, 1.01–1.65; *p* = 0.040), suggesting a potential signal that merits further investigation. This is consistent with recent registry reports from Scandinavia and the United States [15]. On the other hand, cemented THA has been reported to have a higher dislocation rate [4]. Dislocation risk after THA is multifactorial, influenced by surgical approach, head size, original disease, component orientation, and patient factors such as abductor strength and spinopelvic alignment [6, 12, 13, 27, 31]. Although cemented fixation is often chosen in frail elderly patients with reduced muscle tone, which may predispose to increased instability, the widespread use of larger femoral heads and optimized soft tissue repair may limit the direct impact of fixation method on dislocation risk. Because the present analysis was limited to in-hospital outcomes, the relationship between fixation method and long-term complications, including late dislocation and implant survival, cannot be determined. Therefore, the clinical implications of these early differences for long-term outcomes remain uncertain.

The present study demonstrated that cemented fixation was associated with a 24% lower risk of blood transfusion compared with uncemented fixation (OR 0.76; 95% CI, 0.74–0.78). Nils Meißner et al. reported that although cemented and hybrid THA resulted in less blood loss, this did not translate into a lower transfusion rate, and that other confounding factors such as age, comorbidities, and preoperative anemia had a significant impact [18]. Given that perioperative transfusion is a well-known risk factor for postoperative infection, delayed mobilization, and prolonged hospital stay, the decreased transfusion rate associated with cemented fixation may confer additional clinical advantages, particularly in elderly or anemic patients. TXA use was comparable between groups after matching, although a small residual difference remained, suggesting that the observed difference in transfusion rates is unlikely to be explained solely by variation in TXA administration.

Another notable finding was the significantly lower incidence of postoperative DVT in the cemented group (OR 0.79; 95% CI, 0.74–0.84), while no statistically significant differences were observed based on the predefined threshold (*p* < 0.001) in the rates of pulmonary embolism, cardiac events, cerebrovascular complications, or in-hospital mortality. This is in contrast to a report that suggested a slight increase in the incidence of thromboembolism with the use of cement [11, 19]. The mechanism underlying the reduced DVT risk with cemented fixation remains uncertain and cannot be determined from the present database. Although factors such as differences in postoperative pain control or mobilization have been hypothesized, these variables are not captured in the DPC database and therefore cannot be evaluated in this study. Accordingly, this finding should be interpreted as an association rather than a causal relationship, and may be influenced by unmeasured confounding.

In contrast, although no statistically significant difference in in-hospital mortality was observed based on our predefined threshold, the multivariable analysis demonstrated an approximately twofold higher odds of mortality in the cemented group (OR, 1.96; 95% CI, 1.07–3.60; *p* = 0.026). This finding should be interpreted with caution but may represent a potentially clinically relevant signal. One possible explanation is residual confounding, as cemented fixation is more frequently selected for older and more medically complex patients, who inherently have a higher baseline risk of perioperative mortality. In addition, factors such as frailty, functional status, and intraoperative hemodynamic changes, including bone cement implantation syndrome, could not be fully captured in the administrative database. Although historical concerns regarding BCIS have been reported [1], our findings, together with previous large registry studies demonstrating comparable mortality between fixation types [2, 3, 5, 15, 23], these findings do not allow a definitive conclusion regarding the effect of cement use on in-hospital mortality. However, the observed direction and magnitude of the association underscore the importance of careful patient selection and warrant further investigation in studies with more detailed clinical data. Because bone quality is a major determinant of fixation choice and a key source of confounding by indication, we conducted additional age-stratified analyses as a surrogate assessment of bone quality. The consistency of the findings across age strata, together with the results of sensitivity analyses using alternative matching specifications, suggests that the observed associations are unlikely to be fully explained by residual confounding.

This study has several limitations. First, the analysis was limited to patients who underwent THA in hospitals participating in the DPC database, and therefore patients treated in non-reporting institutions were not included. These facilities represent approximately 30% of all general hospital beds in Japan, and their exclusion may have introduced selection bias [34]. Second, although rigorous PS matching was performed, residual confounding from unmeasured variables cannot be excluded. Several procedure-related factors that may influence postoperative outcomes, including implant design, cementing technique, surgical approach, femoral head size, stem geometry, operative time, anesthesia type, bone quality, perioperative management, and surgeon experience, were not available in the DPC database. These variables may affect complications such as dislocation, transfusion requirements, and thromboembolic events. In addition, detailed information on transfusion thresholds and perioperative hemoglobin levels was not available in the DPC database, and variation in institutional transfusion practices may have influenced the observed differences. Therefore, residual confounding related to surgical technique cannot be completely excluded. In particular, although recent studies have highlighted the potential benefit of collared uncemented stems in reducing periprosthetic fracture [8, 16, 24, 25], further research is needed to determine whether the presence or absence of a collar could not be determined in our dataset, which might have affected the results. Third, an important limitation is that the present analysis was restricted to in-hospital outcomes. The DPC database does not capture post-discharge events, and therefore complications occurring after discharge, including late infection, venous thromboembolism, or implant-related complications, could not be evaluated. As a result, the incidence of complications such as periprosthetic joint infection and venous thromboembolism may have been underestimated, particularly given the relatively short length of hospital stay in contemporary practice. In addition, infections defined over a longer postoperative period (e.g., within 6 weeks) could not be assessed. In addition, the DPC database does not provide detailed information on the diagnostic methods for DVT or on thromboprophylaxis strategies, including the type and duration of anticoagulant use or mechanical prophylaxis, which may have influenced the observed results. Prospective studies with longitudinal follow-up are required to clarify the long-term safety and effectiveness of cemented fixation. Fourth, cement use was identified from perioperative medication codes, which may have resulted in some degree of misclassification. The DPC system does not record whether antibiotic-loaded cement was applied, limiting the precision of exposure assessment. Fifth, Bone quality parameters such as bone mineral density and femoral morphology (e.g., Dorr classification) were not available in the administrative database. Because these factors strongly influence the choice between cemented and uncemented fixation, residual confounding related to bone quality may remain even after propensity score matching. Finally, the database lacks detailed clinical data on the severity of comorbidities or complications. Events that cannot be captured by DPC diagnostic codes, such as bone cement implantation syndrome, were not analyzed. Despite these limitations, the present nationwide analysis provides robust real-world evidence that cemented total hip arthroplasty reduces the risks of transfusion and deep vein thrombosis without increasing medical complications or in-hospital mortality.

## Conclusion

In this nationwide PS matched analysis using the Japanese DPC database, cemented THA was associated with a lower risk of periprosthetic fracture, blood transfusion, and DVT, without increasing the rates of infection, dislocation, medical complications, or in-hospital mortality. These findings suggest that cemented fixation may be associated with favorable short-term outcomes in selected patients. In the context of Japan’s aging population and the current predominance of uncemented fixation, careful reconsideration of cemented techniques may help optimize outcomes and patient safety in total hip arthroplasty.

## Supplementary Information

Below is the link to the electronic supplementary material.

Supplementary file1 (DOCX 17 KB)

Supplementary file2 (DOCX 17 KB)

Supplementary file3 (DOCX 17 KB)

Supplementary file4 (DOCX 17 KB)

Supplementary file5 (DOCX 17 KB)

Supplementary file6 (DOCX 17 KB)

## Data Availability

No datasets were generated or analysed during the current study.
