# Supplementary material for: Cemented total hip arthroplasty reduces early complications: a Japanese nationwide propensity-matched study
Source: Arch Orthop Trauma Surg. 2026 May 2;146(1):168. doi: 10.1007/s00402-026-06328-x (PMC13135592; doi:10.1007/s00402-026-06328-x)
Supplement: Supplementary file 1 — Supplementary file1 (DOCX 17 KB) [file 402_2026_6328_MOESM1_ESM.docx]

| **Supplementary Table S1. Age-stratified multivariable logistic regression analysis of surgical complications in the propensity score–matched cohort (65–74 years)** | | | | | | | | | | |
| --- | --- | --- | --- | --- | --- | --- | --- | --- | --- | --- |
| Complications |  |  |  | Univariate analysis |  |  |  | Multivariable analysis |  |  |
|  | n |  | OR | 95% CI | *P-value* |  | OR | 95% CI | χ2 statics | *P-value* |
| Dislocation | 243 |  | 1.111 | 0.863 to 1.430 | 0.440 |  | 1.396 | 0.977 to 2.000 | 3.360 | 0.067 |
| Infection | 264 |  | 0.780 | 0.611 to 0.996 | 0.048 |  | 0.777 | 0.607 to 0.995 | 4.029 | 0.045 |
| Periprosthetic fracture | 107 |  | 0.319 | 0.205 to 0.497 | < 0.001 |  | 0.355 | 0.225 to 0.561 | 22.43 | < 0.001 |
| Wound dehiscence | 41 |  | 0.950 | 0.515 to 1.753 | 0.877 |  | 0.981 | 0.528 to 1.823 | 0.004 | 0.953 |
| Mechanical loosening | 21 |  | 0.748 | 0.315 to 1.775 | 0.523 |  | 0.837 | 0.347 to 2.019 | 0.158 | 0.631 |
| Transfusion | 22364 |  | 0.695 | 0.667 to 0.724 | < 0.001 |  | 0.696 | 0.668 to 0.725 | 303.5 | < 0.001 |
| Reoperation | 479 |  | 0.845 | 0.705 to 1.013 | 0.073 |  | 0.822 | 0.629 to 1.072 | 2.103 | 0.147 |
| P-values of < 0.001 are considered significant by the χ2 test | | | | |  |  |  |  |  |  |
| OR; Odds Ratio, CI; Confidence Interval. | |  |  |  |  |  |  |  |  |  |
