# Supplementary material for: Cemented total hip arthroplasty reduces early complications: a Japanese nationwide propensity-matched study
Source: Arch Orthop Trauma Surg. 2026 May 2;146(1):168. doi: 10.1007/s00402-026-06328-x (PMC13135592; doi:10.1007/s00402-026-06328-x)
Supplement: Supplementary file 2 — Supplementary file2 (DOCX 17 KB) [file 402_2026_6328_MOESM2_ESM.docx]

| **Supplementary Table S2. Age-stratified multivariable logistic regression analysis of medical complications in the propensity score–matched cohort (65–74 years)** | | | | | | | | | | |
| --- | --- | --- | --- | --- | --- | --- | --- | --- | --- | --- |
| Complications |  |  |  | Univariate analysis |  |  |  | Multivariable analysis |  |  |
|  | n |  | OR | 95% CI | *P-value* |  | OR | 95% CI | χ2 statics | *P-value* |
| Hospital-acquired pneumonia | 52 |  | 1.258 | 0.727 to 2.175 | 0.488 |  | 1.315 | 0.757 to 2.283 | 0.953 | 0.329 |
| DVT | 1986 |  | 0.811 | 0.741 to 0.888 | < 0.001 |  | 0.813 | 0.743 to 0.891 | 19.96 | < 0.001 |
| PE | 73 |  | 0.657 | 0.411 to 1.050 | 0.080 |  | 0.668 | 0.417 to 1.071 | 2.867 | 0.090 |
| Cardiac event | 1 |  | - | - | 1.000 |  | 0.000 | - | 1.370 | 2.418 |
| Cerebrovascular event | 60 |  | 0.619 | 0.368 to 1.043 | 0.072 |  | 0.612 | 0.363 to 1.032 | 3.496 | 0.062 |
| Acute renal falure | 6 |  | 0.499 | 0.091 to 2.722 | 0.453 |  | 0.492 | 0.090 to 2.687 | 0.712 | 0.399 |
| Sepsis | 211 |  | 0.755 | 0.575 to 0.992 | 0.045 |  | 0.759 | 0.578 to 0.998 | 3.928 | 0.048 |
| Mortality during hospitalization | 11 |  | 1.197 | 0.365 to 3.922 | 1.000 |  | 1.290 | 0.388 to 4.293 | 0.173 | 0.677 |
| P-values of < 0.001 are considered significant by the χ2 test | | | | |  |  |  |  |  |  |
| OR; Odds Ratio, CI; Confidence Interval, DVT; Deep Vein Thrombosis, PE; Pulmonary Embolism. | | | | | | | | |  |  |
