# Supplementary material for: Cemented total hip arthroplasty reduces early complications: a Japanese nationwide propensity-matched study
Source: Arch Orthop Trauma Surg. 2026 May 2;146(1):168. doi: 10.1007/s00402-026-06328-x (PMC13135592; doi:10.1007/s00402-026-06328-x)
Supplement: Supplementary file 3 — Supplementary file3 (DOCX 17 KB) [file 402_2026_6328_MOESM3_ESM.docx]

| **Supplementary Table S3. Age-stratified multivariable logistic regression analysis of surgical complications in the propensity score–matched cohort (75–84 years)** | | | | | | | | |  | |  | |  |
| --- | --- | --- | --- | --- | --- | --- | --- | --- | --- | --- | --- | --- | --- |
| Complications |  |  |  | Univariate analysis |  |  |  | Multivariable analysis | |  | |  | |
|  | n |  | OR | 95% CI | *P-value* |  | OR | 95% CI | | χ2 statics | | *P-value* | |
| Dislocation | 266 |  | 0.961 | 0.755 to 1.223 | 0.759 |  | 1.088 | 0.760 to 1.557 | | 0.212 | | 0.645 | |
| Infection | 255 |  | 0.879 | 0.687 to 1.125 | 0.315 |  | 0.881 | 0.685 to 1.133 | | 0.981 | | 0.322 | |
| Periprosthetic fracture | 119 |  | 0.385 | 0.257 to 0.575 | < 0.001 |  | 0.403 | 0.264 to 0.617 | | 19.19 | | < 0.001 | |
| Wound dehiscence | 30 |  | 1.006 | 0.491 to 2.058 | 1.000 |  | 1.053 | 0.514 to 2.159 | | 0.020 | | 0.888 | |
| Mechanical loosening | 19 |  | 0.747 | 0.315 to 1.773 | 0.523 |  | 0.821 | 0.323 to 2.084 | | 0.174 | | 0.676 | |
| Transfusion | 16765 |  | 0.814 | 0.778 to 0.852 | < 0.001 |  | 0.814 | 0.778 to 0.852 | | 78.5 | | < 0.001 | |
| Reoperation | 473 |  | 0.829 | 0.690 to 0.994 | 473 |  | 0.918 | 0.690 to 1.222 | | 0.346 | | 0.556 | |
| P-values of < 0.001 are considered significant by the χ2 test | | | | |  |  |  |  | |  | |  | |
| OR; Odds Ratio, CI; Confidence Interval. | |  |  |  |  |  |  |  | |  | |  | |
