# Supplementary material for: Cemented total hip arthroplasty reduces early complications: a Japanese nationwide propensity-matched study
Source: Arch Orthop Trauma Surg. 2026 May 2;146(1):168. doi: 10.1007/s00402-026-06328-x (PMC13135592; doi:10.1007/s00402-026-06328-x)
Supplement: Supplementary file 4 — Supplementary file4 (DOCX 17 KB) [file 402_2026_6328_MOESM4_ESM.docx]

| **Supplementary Table S4. Age-stratified multivariable logistic regression analysis of medical complications in the propensity score–matched cohort (75–84 years)** | | | | | | | | | | |
| --- | --- | --- | --- | --- | --- | --- | --- | --- | --- | --- |
| Complications |  |  |  | Univariate analysis |  |  |  | Multivariable analysis |  |  |
|  | n |  | OR | 95% CI | *P-value* |  | OR | 95% CI | χ2 statics | *P-value* |
| Hospital-acquired pneumonia | 64 |  | 0.945 | 0.578 to 1.543 | 0.801 |  | 0.908 | 0.554 to 1.491 | 0.145 | 0.704 |
| DVT | 1613 |  | 0.756 | 0.683 to 0.836 | < 0.001 |  | 0.756 | 0.684 to 0.837 | 29.38 | < 0.001 |
| PE | 69 |  | 0.870 | 0.541 to 1.396 | 0.630 |  | 0.837 | 0.519 to 1.348 | 0.539 | 0.463 |
| Cardiac event | 10 |  | 2.347 | 0.607 to 9.079 | 0.225 |  | 1.866 | 0.462 to 7.542 | 0.810 | 0.368 |
| Cerebrovascular event | 103 |  | 1.247 | 0.845 to 1.840 | 0.279 |  | 1.252 | 0.847 to 1.849 | 1.280 | 0.258 |
| Acute renal failure | 8 |  | 0.603 | 0.144 to 2.525 | 1.000 |  | 0.629 | 0.150 to 2.639 | 0.414 | 0.52 |
| Sepsis | 191 |  | 0.823 | 0.618 to 1.095 | 0.192 |  | 0.830 | 0.623 to 1.105 | 1.641 | 0.2 |
| Mortality during hospitalization | 23 |  | 2.852 | 1.124 to 7.235 | 0.022 |  | 2.719 | 1.054 to 7.013 | 4.800 | 0.029 |
| P-values of < 0.001 are considered significant by the χ2 test | | | | |  |  |  |  |  |  |
| OR; Odds Ratio, CI; Confidence Interval, DVT; Deep Vein Thrombosis, PE; Pulmonary Embolism. | | | | | | | | |  |  |
