# Supplementary material for: Cemented total hip arthroplasty reduces early complications: a Japanese nationwide propensity-matched study
Source: Arch Orthop Trauma Surg. 2026 May 2;146(1):168. doi: 10.1007/s00402-026-06328-x (PMC13135592; doi:10.1007/s00402-026-06328-x)
Supplement: Supplementary file 5 — Supplementary file5 (DOCX 17 KB) [file 402_2026_6328_MOESM5_ESM.docx]

| **Supplementary Table S5. Age-stratified multivariable logistic regression analysis of surgical complications in the propensity score–matched cohort (≥85 years)** | | | | | | | | | | |
| --- | --- | --- | --- | --- | --- | --- | --- | --- | --- | --- |
| Complications |  |  |  | Univariate analysis |  |  |  | Multivariable analysis |  |  |
|  | n |  | OR | 95% CI | *P-value* |  | OR | 95% CI | χ2 statics | *P-value* |
| Dislocation | 52 |  | 1.876 | 1.057 to 3.330 | 0.036 |  | 1.862 | 0.702 to 4.944 | 1.575 | 0.210 |
| Infection | 40 |  | 0.59 | 0.310 to 1.122 | 0.014 |  | 0.582 | 0.300 to 1.130 | 2.641 | 0.104 |
| Periprosthetic fracture | 25 |  | 0.554 | 0.244 to 1.256 | 0.165 |  | 0.549 | 0.235 to 1.284 | 1.985 | 0.159 |
| Wound dehiscence | 8 |  | 0.987 | 0.247 to 3.952 | 1.000 |  | 1.045 | 0.258 to 4.222 | 0.004 | 0.951 |
| Mechanical loosening | 0 |  | - | - | - |  | - | - | - | - |
| Transfusion | 2792 |  | 0.959 | 0.862 to 1.068 | 0.460 |  | 0.955 | 0.857 to 1.063 | 0.7 | 0.968 |
| Reoperation | 78 |  | 1.352 | 0.860 to 2.126 | 0.21 |  | 1.023 | 0.455 to 2.300 | 0.001 | 0.955 |
| P-values of < 0.001 are considered significant by the χ2 test | | | | |  |  |  |  |  |  |
| OR; Odds Ratio, CI; Confidence Interval. | |  |  |  |  |  |  |  |  |  |
