# Supplementary material for: Cemented total hip arthroplasty reduces early complications: a Japanese nationwide propensity-matched study
Source: Arch Orthop Trauma Surg. 2026 May 2;146(1):168. doi: 10.1007/s00402-026-06328-x (PMC13135592; doi:10.1007/s00402-026-06328-x)
Supplement: Supplementary file 6 — Supplementary file6 (DOCX 17 KB) [file 402_2026_6328_MOESM6_ESM.docx]

| **Supplementary Table S6. Age-stratified multivariable logistic regression analysis of medical complications in the propensity score–matched cohort (≥85 years)** | | | | | | | | | | | | |
| --- | --- | --- | --- | --- | --- | --- | --- | --- | --- | --- | --- | --- |
| Complications |  |  |  | Univariate analysis |  |  | |  | Multivariable analysis |  | |  |
|  | n |  | OR | 95% CI | *P-value* | |  | OR | 95% CI | | χ2 statics | *P-value* |
| Hospital-acquired pneumonia | 26 |  | 0.987 | 0.457 to 2.134 | 1.000 |  | | 0.921 | 0.422 to 2.008 | 0.043 | | 0.835 |
| DVT | 283 |  | 0.831 | 0.653 to 1.056 | 0.143 |  | | 0.824 | 0.647 to 1.049 | 2.483 | | 0.115 |
| PE | 18 |  | 1.979 | 0.742 to 5.281 | 0.237 |  | | 2.014 | 0.751 to 5.398 | 2.053 | | 0.152 |
| Cardiac event | 3 |  | 1.975 | 0.179 to 21.80 | 1.000 |  | | 1.960 | 0.178 to 21.63 | 0.320 | | 0.572 |
| Cerebrovascular event | 25 |  | 1.484 | 0.665 to 3.309 | 0.424 |  | | 1.491 | 0.667 to 3.334 | 0.968 | | 0.325 |
| Acute renal falure | 6 |  | 4.944 | 0.577 to 42.35 | 0.219 |  | | 4.713 | 0.545 to 40.78 | 2.631 | | 0.105 |
| Sepsis | 33 |  | 0.492 | 0.238 to 1.016 | 0.056 |  | | 0.493 | 0.238 to 1.021 | 3.851 | | 0.050 |
| Mortality during hospitalization | 14 |  | 1.780 | 0.596 to 5.318 | 0.029 |  | | 1.682 | 0.546 to 5.177 | 0.849 | | 0.357 |
| P-values of < 0.001 are considered significant by the χ2 test | | | | |  |  | |  |  |  | |  |
| OR; Odds Ratio, CI; Confidence Interval, DVT; Deep Vein Thrombosis, PE; Pulmonary Embolism. | | | | | | | | | |  | |  |
